# Exogenous Hydrogen Sulfide Activates PI3K/Akt/eNOS Pathway to Improve Replicative Senescence in Human Umbilical Vein Endothelial Cells

**DOI:** 10.1155/2023/7296874

**Published:** 2023-04-06

**Authors:** Haiming Niu, Jianwei Li, Hongkai Liang, Guishen Wu, Miaolian Chen

**Affiliations:** Department of Critical Care Medicine, Zhongshan People's Hospital, Zhongshan 528400, China

## Abstract

**Background:**

Endothelial cell senescence is one of the key mechanistic factors in the pathogenesis of atherosclerosis. In terms of molecules, the phosphatidylinositol 3-kinase/protein kinase B/endothelial nitric oxide synthase (PI3K/Akt/eNOS) signaling plays an important role in the prevention and control of endothelial cell senescence, while hydrogen sulfide (H2S) improves the induced precocious senescence of endothelial cells through the PI3K/Akt/eNOS pathway. Comparatively, replicative senescence in endothelial cells is more in line with the actual physiological changes of human aging. This study aims to investigate the mechanism by which H2S improves endothelial cell replicative senescence and the involvement of the PI3K/Akt/eNOS pathway.

**Methods:**

we established a model of replicative senescence in human umbilical vein endothelial cells (HUVECs) and explored the effect of 200 *μ*mol/L sodium hydrosulfide (NaHS; a donor of H2S) on senescence, which was determined by cell morphology, the expression level of plasminogen activator inhibitor 1 (PAI-1), and the positive rate of senescence-associated *β*-galactosidase (SA-*β*-Gal) staining. Cell viability was detected by MTT assay to evaluate the effect of NaHS and the PI3K inhibitor, LY294002. Meanwhile, the protein expression of PI3K, Akt, p-Akt, and eNOS in endothelial cells of each group was detected by Western blot.

**Results:**

the replicative senescence model was established in HUVECs at the passage of 16 cumulative cell population doubling values (CPDL). Treatment with NaHS not only significantly reduced the expression of PAI-1 and the positive rate of SA-*β*-Gal in HUVEC's replicative senescence model but also notably increased the expression of PI3K, p-Akt, p-eNOS, and the content of nitric oxide(NO). However, the effects of NaHS on the expression of the pathway and the content of NO in HUVECs were abolished when LY294002 specifically inhibited PI3K.

**Conclusion:**

NaHS improves the replicative senescence of HUVECs with the contribution of the PI3K/Akt/eNOS pathway.

## 1. Introduction

With the world slipping into an aging society, the morbidity and mortality of cardiovascular disease are on the rise. It is estimated that the number of people suffering from cardiovascular disease in China reaches 330 million, which contributes to the leading cause of death among urban and rural residents [1]. Continued research into the mechanisms of the development of cardiovascular disease has gradually concluded that endothelial cell senescence is the significant driving cascade [2]; moreover, age-related endothelial cell senescence is an important factor leading to the poor prognosis of cardiovascular disease [3]. Endothelial cell senescence includes replicative senescence and precocious senescence. In comparison, endothelial cell replicative senescence is linked with the gradual shortening of telomeres during routine cell division and the subsequent genomic instability, which is more in line with the actual physiological changes during aging. Therefore, improving the replicative senescence of endothelial cells is of great significance for the prevention and treatment of atherosclerosis [4].

It has been confirmed that the phosphatidylinositol 3-kinase/protein kinase B (PI3K/Akt) pathway regulates cell proliferation [5, 6] and mediates the phosphorylation of endothelial nitric oxide synthase (eNOS), thereby affecting the content of nitric oxide (NO) [7] to play an antiaging effect. In vitro studies have confirmed that the PI3K/Akt/eNOS pathway protects endothelial cells from the damage of reactive oxygen species (ROS) [8]. In recent years, there has emerged more and more studies on the PI3K/Akt/eNOS signaling in the premature aging model of endothelial cells. For example, it rescued hydrogen peroxide-induced endothelial cell aging [9] as well as protecting and ameliorating endothelial cell injury induced by glucose [10]. Therefore, the activation of the PI3K/Akt/eNOS signaling pathway is the key to precise targeting in the regulation of endothelial cell function and might further contribute to the prevention of and treatment of human arterial vascular disease. Notably, the mechanism by which the PI3K/Akt/eNOS axis functions in replicative endothelial cell senescence remains unclear.

Hydrogen sulfide (H_2_S) is the third gaseous signaling molecule in the human body [11], which can reverse the formation of atherosclerotic plaques [12]. Piling studies have confirmed the effect of H_2_S in resisting the induced senescence of endothelial cells and restoring the function of damaged endothelial cells [9, 10, 13], whose detailed mechanism has not been fully elucidated, has not mentioned the replicative senescence model of endothelial cells. In this study, we established a replicative senescence model of HUVECs that duplicates the physiological condition of natural aging to investigate the effect of exogenous H_2_S on the replicative senescence of endothelial cells and explored the effect and mechanism of exogenous H_2_S in decelerating the replicative senescence of endothelial cell from the perspective of the PI3K/Akt/eNOS pathway.

## 2. Materials and Methods

### 2.1. Primary Extraction, Culture, and Cell Age Calculation of HUVECs

This study was approved by the Ethics Committee of Zhongshan Hospital Affiliated to Sun Yat-Sen University and adhered to the principles in the Helsinki Declaration. The isolation and culture of primary HUVECs referred to the current classical method [14]. Briefly, the umbilical cord of a healthy neonate was rinsed with PBS three times under aseptic conditions, followed by digestion with 0.1% collagenase I (GIBCO) for 15 minutes. The mixture was centrifuged at 300 × *g* for 7 minutes to discard the supernatant. Pellets were resuspended in complete medium comprised of 20% FBS (GIBCO), 20% SFM (GIBCO), and 60 *μ*g/mL ECGS (BD), and inoculated in a T25 culture flask precoated with 0.2% gelatin. After one day of culture in a sterile incubator at 37°C and supplemented with 5% CO_2_, cells were washed with PBS in the ultraclean workbench to remove those endothelial cells that were lacking in adherent growth and the residual erythrocytes, and then were returned to the incubator. Cells were digested at a confluency of more than 90% with EDTA-containing 0.05% trypsin on an ultraclean workbench and transferred to a T25 culture flask for subsequent culture. The “age” of cells is represented by the cumulative cell population doubling level (CPDL). The CPDL was calculated as follows: population doublings (PD) = (log10F-log10I)/0.301 (F: the number of cells at the passage; I: the number of cells seeded last time), and PD calculated at each passage was added to the previous one to obtain the CPDL. CPDL reflects the “age” of cells more objectively and is more reproducible than passage in experiments. In recent years, platelet endothelial cell adhesion molecule-1 (CD31) has been widely used to identify endothelial cells. Therefore, HUVECs at passage 1 were digested and collected to examine the cell surface marker CD31 under an inverted microscope in the central laboratory of our hospital.

### 2.2. Establishment of Senescent Model and Cell Grouping

#### 2.2.1. Establishment of Replicative Senescence Model

Senescence-related phenotypes, including cell morphology, positive rate of senescence-associated* β*-galactosidase (SA-*β*-Gal) staining (KeyGEN BioTECH Co., Ltd.) and plasminogen activator inhibitor 1 (PAI-1) protein expression in different CPDL stages of cells were monitored. Cells in a certain CPDL stage with positive and stable senescent indicators were recognized as senescence models for the following experiments.

#### 2.2.2. Cell Grouping

In order to explore whether hydrogen sulfide can improve the replicative senescence of endothelial cells, we performed experiments on three groups: the control group (CPDL 2 HUVECs), the replicative senescence model group, and the sodium hydrosulfide (NaHS; a donor of H_2_S) intervention group (replicative senescence model treated with 200 *μ*mol/L NaHS for 24 hours; NaHS was purchased from Sigma company).

In order to confirm the hypothesis that hydrogen sulfide improved the replicative senescence of endothelial cells through PI3K/Akt/eNOS signaling, we designed four groups for the mechanistic investigation: control group (CPDL 2 HUVECs), replicative senescence model group, NaHS intervention group (replicative senescence model treated with 200 *μ*mol/L NaHS for 24 hours), and PI3K-blocked group (replicative senescence model treated with 10 *μ*mol/L PI3K inhibitor, LY294002 for 1 hour, and 200 *μ*mol/L NaHS for another 24 hours). According to relative studies, the PI3K/Akt/eNOS pathway is suppressed in endothelial cell injury, aging, and other models, and LY294002 treatment exerted little effect, in that we didn't included the RS + LY group in this study [8, 10]. LY294002 was purchased from Promega.

### 2.3. Cell Viability Assay

Methyl thiazolyl tetrazolium (MTT) assay was applied to detect the effect of hydrogen sulfide at different concentrations on cell viability with the help of MTT kit (Nanjing Jiancheng Bioengineering Institute). HUVECs were seeded on 96-well plates with 4000 cells per well and five replicates for each treatment. Cells were treated after adherence and divided into 5 groups: the zeroing group, the control group, the NaHS intervention group, the LY294002 treatment group, and LY294002-pretreated NaHS intervention group. After 24 hours of treatment, cells were incubated with 20 *μ*L of 5 mg/mL MTT for 4 hours; then the medium was removed, and 150 *μ*L of DMSO was added to each well. The plates were covered with tin foil and placed on a shaker for 15 minutes to mix thoroughly. Finally, the absorbance value (OD) at 490 nm of each well was read with a microplate reader.

### 2.4. Western Blot

The protein extraction was operated strictly in accordance with the instructions of the whole protein extraction kit of Jiangsu KeyGEN BioTECH Co., Ltd. Briefly, every 700 *μ*L lysate was supplemented with 7 *μ*L phosphatase inhibitor, 0.7 *μ*L protease inhibitor, and 3.5 *μ*L PMSF to prepare protein extraction solution. Cells were washed twice with PBS, lysed in protein extraction solution on ice for 10 minutes, detached from culture plates with cell scrapers, and then collected into a prelabeled and precooled EP tube with a pipette. EP tubes were shacked on ice for 15 minutes for mixing and thorough lysing, followed by centrifuge at 14,000 × *g* upon 4°C for 15 minutes. Supernatants were subjected to protein concentration determination using BCA, and aliquoted to store in a −80°C refrigerator for further experiments.

The total amount of 20 *μ*g of protein was loaded in each lane and separated by SDS-polyacrylamide gel electrophoresis and electrotransferred to a polyvinylidene difluoride (PVDF) membrane (Merck Millipore), which was subsequently blocked with 5% skim milk (Yili) at room temperature for 1 h, and incubated with the following primary antibodies: PAI-1 mouse antibody (1 : 1000; Santa Cruz), PI3K rabbit antibody (1 : 1000; Cell Signaling Technology), eNOS rabbit antibody (1 : 1000, Cell Signaling Technology), p-eNOS rabbit antibody (1 : 1000, Cell Signaling Technology), Akt rabbit antibody (1 : 1000; Cell Signaling Technology), p-Akt mouse antibody (1 : 2000; Cell Signaling Technology), and GAPDH rabbit antibody (1 : 10000; Proteintech). Incubation was performed on a shaker in a 4°C refrigerator overnight; the primary antibody was removed the next day and rinsed with TBST (Tris 50 mmol/L, NaCl 100 mmol/L, pH 7.5, containing 1% Tween20; MBCHEM) for 5 minutes and 3 times, and then PVDF membranes were incubated in secondary antibodies (Boster), including horseradish-labeled goat antimouse IgG (1 : 10000) and horseradish-labeled goat antirabbit IgG (1 : 10000), at room temperature for 1 hour. The secondary antibodies were discarded, followed by three washes with 1 × TBST on a shaker. ECL kit (Merck Millipore) was utilized to develop, and protein bands were analyzed by ImageJ software.

### 2.5. Determination of NO Content in Cell Supernatant

NO content detection was performed according to the NO kit instructions of the Nanjing Jiancheng Bioengineering Institute. Briefly, endothelial cell supernatants were collected, and every 0.1 mL sample was added to 0.4 mL of mixed reagent (reagent 1: reagent 2 = 1 : 1), and bathed at 37°C for 60 minutes. Samples were further mixed with 0.2 mL reagent 3 and 0.1 mL reagent 4, vibrated on a vortex for 30 seconds, and then placed at room temperature for 40 minutes, followed by centrifugation at 1000 × *g* for 10 minutes; 0.5 mL of the supernatant from each sample was added to 0.6 mL of chromogenic reagent and mixed. After placing at room temperature for 10 minutes, the absorbance values at 550 nm of each group were measured.

### 2.6. Statistical Processing

SPSS 21.0 analysis software was used for statistic description and examination. Quantitative data were expressed as mean ± standard deviation ($\bar{x}\pm s$). Measurement data of multiple groups were confirmed to be normally distributed with homogenous variance and compared using a single one-way analysis of variance (one-way ANOVA) to verify the differences among groups; then pairwise comparison was applied with the LSD-t method when the difference was statistically significant among groups. *P* < 0.05 was considered to be statistically significant.

## 3. Results

### 3.1. Identification of Endothelial Cells

**Figure 1:**
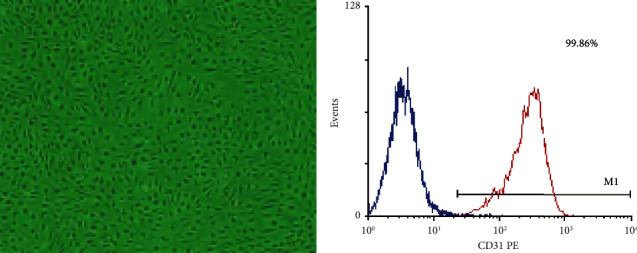
Identification of HUVECs. (a) Optical microscopy images of early HUVECs (40×). (b) Detection of CD31 on HUVECs surface by flow cytometry.

HUVECs initially digested from the umbilical vein were circular when observed under an inverted microscope. Cells were completely adherent for 24 hours after inoculation and became cells were polygonal, with abundant cytoplasm, clear nuclei, and frequent mitotic phases at the early stage. After infusion, cells presented a typical cobblestone-like arrangement (Figure 1(a)). In 48–72 hours, the primary cells were about 90% confluent and passed at a ratio of 1 : 2 or 1 : 3. Cells of passage 1 exhibited 99.86% positives of the cell surface marker CD31 (Figure 1(b)), which was consistent with the characteristics of endothelial cells.

### 3.2. Establishment of the Senescence Model

**Figure 2:**
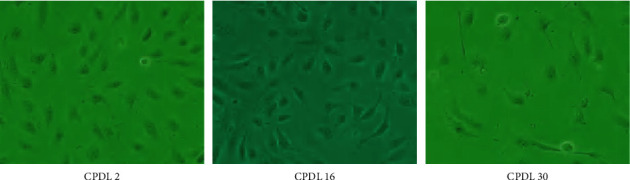
Morphology of HUVECs at different CPDLs under optical microscopes (200×).

**Figure 3:**
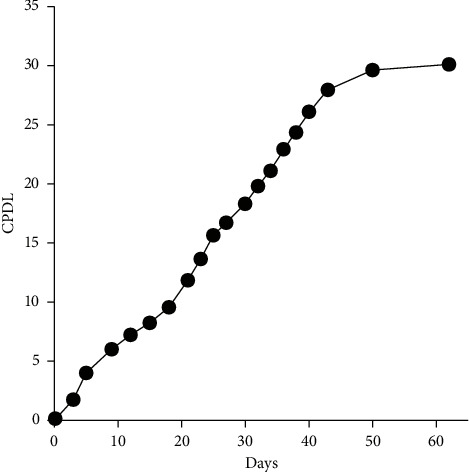
Proliferation curve of HUVECs at different CPDLs.

**Figure 4:**
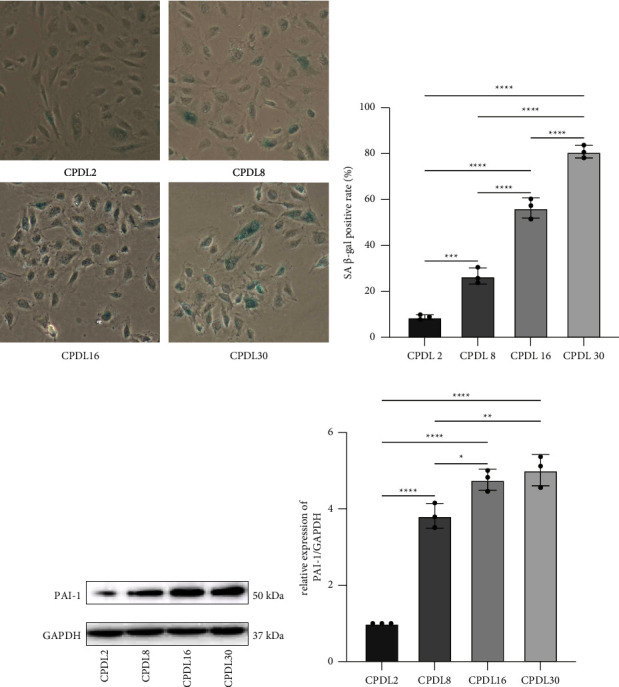
Establishment of replicative senescence model in HUVECs. SA-*β*-Gal staining (a) and the statistics (b) of HUVECs at different CPDLs (100×). Senescent cells were stained blue. The CPDL 8, 16 and 30 groups showed a significant increase SA-*β*-Gal level as compared with the CPDL 2 group. *n* = 3 per group. ^*∗∗∗*^*P* < 0.001, ^*∗∗∗∗*^*P* < 0.0001 by LSD-t test after ANOVA. The expression of PAI-1 of HUVECs at different CPDLs as detected by western blot (c) and its statistics (d) The CPDL8,16, and 30 groups showed a significant increase of PAI- 1 protein expression compared to the CPDL 2 group. *n* = 3/group. ^*∗*^*P* < 0.05, ^*∗∗*^*P* < 0.01, ^*∗∗∗∗*^*P* < 0.0001 as detected by LSD-t test after ANOVA.

The replicative senescence model took a longer time to simulate but was closer to the physiological course than induced senescence. The objective “age” of HUVECs was quantified by CPDL. With the accumulation of CPDL, cells deformed as the edges blurred and long antennae emerged, accompanied by decreased cell adherence. In detail, cell morphology began to change at CPDL 16 and nearly all cells deformed at CPDL 30, distinguishing notably with cells at CPDL 2 with an increased proportion of dead cells (Figure 2). With CPDL as the vertical axis and passage time as the horizontal axis, the growth curve of the obtained HUVECs demonstrated that at CPDL 26 to 30, cell proliferation rate significantly slowed down and ultimately stagnated (Figure 3). Previous literature established a replicative senescence model based on the passage of 4 HUVECs [15], which was approximately CPDL 6–8 in our model. Therefore, we choose CPDL2, 8, 16, and 30 for the following experiment. Meanwhile, SA-*β*-Gal staining of HUVECs manifested that CPDL 2 cells were scarcely positive for SA-*β*-Gal, while CPDL 8 were partly blue-stained and most cells at CPDL 16 were deformed. By CPDL 30, cells were deformed and markedly reduced in number, exhibiting a complete positive for SA-*β*-Gal staining (Figures 4(a) and 4(b)). In order to monitor the dynamic changes of cell senescence, we detected the expression of PAI-1 protein in each group by western blot and found that PAI-1 expression was elevated from CPDL 2 to CPDL 8 to CPDL 16 to CPDL 30, consistent with SA-*β*-Gal staining (Figures 4(c) and 4(d)). Thus, the senescence phenotype appeared in CPDL 8 cells and stabilized at CPDL 16. As cell proliferation stagnated when CPDL reached 26 to 30, HUVECs of CPDL 16∼26 are eligible for the replicative senescence model, but CPDL 16 was chosen for follow-up experiments to save time costs.

### 3.3. Effects of NaHS and LY294002 on Endothelial Cell Growth

**Figure 5:**
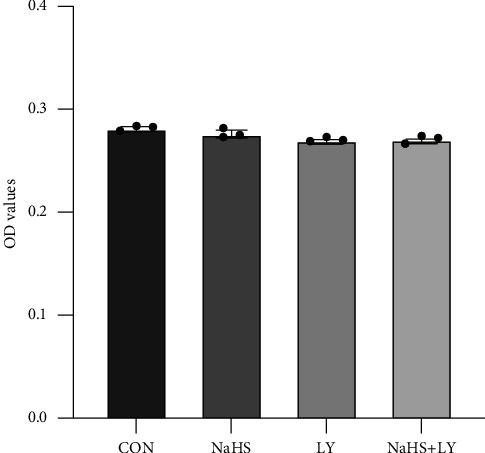
Impact of NaHS and LY294002 on HUVECs growth. No statistical significances were observed among groups. CON: control groups, HUVECs without any treatment. NaHS: HUVECs exposed to 200 *μ*mol/L NaHS. LY: HUVECs exposed to 10 *μ*mol/L LY294002. NaHS + LY: HUVECs exposed to 200 *μ*mol/L NaHS and 10 *μ*mol/L LY294002 simultaneously.

Our previous study confirmed that HUVECs pretreated with 50 *μ*mol/L, 100 *μ*mol/L, and 200 *μ*mol/L of NaHS-for 24 hours were resistant to H_2_O_2_-induced cell senescence in a concentration-dependent manner [9, 16]. In order to exclude the effects of NaHS and LY294002 on the growth of HUVECs, we first performed the MTT assay (Figure 5) and discovered that incubation with 200 *μ*mol/L NaHS for 24 hours, pretreatment by 10 *μ*mol/L LY294002 for 1 hour, or the combination of both exerted little effect on cell viability as compared with the blank control group (*p* > 0.05). Therefore, NaHS at a concentration of 200 *μ*mol/L or LY294002 at 10 *μ*mol/L exerted little influence on HUVECs' proliferation, and we chose 200 *μ*mol/L NaHS for the following experiment.

### 3.4. Effects of NaHS on Replicative Senescence of Endothelial Cells

**Figure 6:**
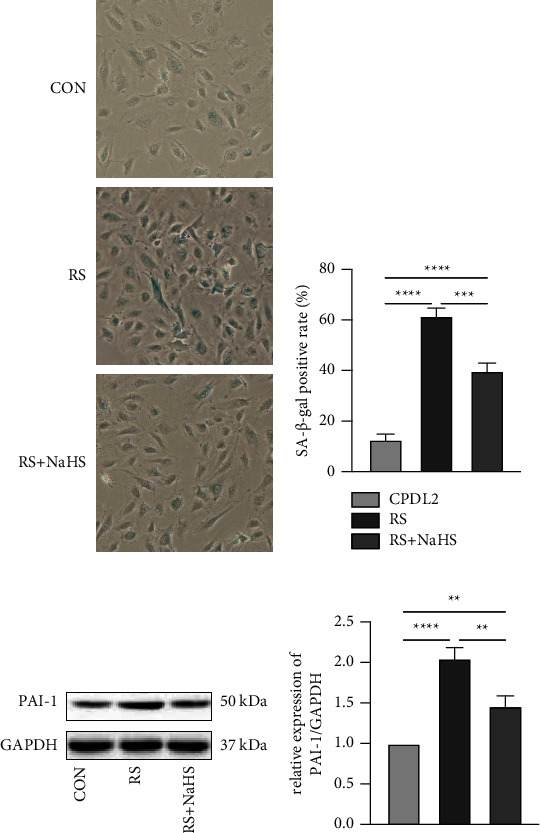
Impact of NaHS on replicative senescence of HUVECs. SA-*β*-Gal staining (a) and the statistics (b) of HUVECs in each group (100×). Senescent cells were stained blue. *n* = 3 per group. ^*∗∗∗*^*P* < 0.001, ^*∗∗∗∗*^*P* < 0.0001 by LSD-t test after ANOVA. The expression of PAI-1 of HUVECs in different groups as detected by western blot (c) and its statistics (d) n = 3 per group. ^*∗∗*^*P* < 0.01, ^*∗∗∗∗*^*P* < 0.0001 as examined by LSD-t test after ANOVA. CON: control group, HUVECs at CPDL2; RS: replicative senescence group, HUVECs at CPDL16; RS + NaHS: HUVECs at CPDL16 and exposed to 200 *μ*mol/L NaHS for 24 hours.

Next, we continued to explore the influence of NaHS on the model. Replicative senescent cells were treated with 200 *μ*mol/L NaHS for 24 hours and subjected to SA-*β*-Gal staining and protein detection. As shown in Figures 6(a) and 6(b), replicative senescence cells featured a high positive rate of SA-*β*-Gal staining in comparison with the control group (CPDL2 cells), which was rescued by NaHS intervention. Similarly, NaHS treatment reduced the expression of PAI-1 in replicative senescent cells (Figures 6(c) and 6(d)). The abovementioned results suggest that NaHS improved the replicative senescence of endothelial cells.

### 3.5. Effects of NaHS Interference on the PI3K/Akt/eNOS Pathway

**Figure 7:**
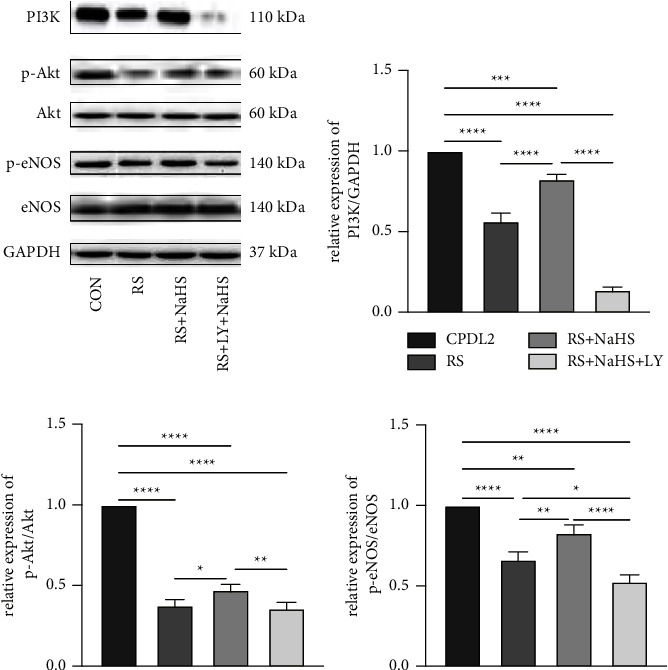
NaHS activated PI3K/Akt/eNOS signaling to alleviate replicative senescence of HUVECs. (a) Western blot analysis and statistics of p-PI3K (b), p-Akt (c), and p-eNOS (d) ^*∗*^*P* < 0.05, ^*∗∗*^*P* < 0.01 as examined by LSD-t test after ANOVA. CON: Control group, HUVECs at CPDL2; RS: replicative Senescence group, HUVECs at CPDL16; RS + NaHS: HUVECs at CPDL16 and exposed to 200 *μ*mol/L NaHS for 24 hours. RS + NaHS + LY:HUVECs at CPDL16 and exposed to 10 *μ*mol/L LY294002 prior to treatment of 200 *μ*mol/L NaHS for 24 hours.

**Figure 8:**
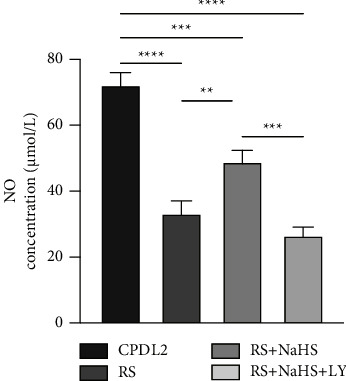
Effect of NaHS pre-treatment on NO concentration in HUVECs. Intracellular NO production was assessed using the nitric oxide fluorometric assay kit and the effects of NaHS on NO concentration in the indicated groups were compared in each group. *n* = 3 per group. ^*∗*^*P* < 0.05, ^*∗∗*^*P* < 0.01 by LSD-t test after ANOVA. CON: control group, HUVECs at CPDL2; RS: replicative senescence group, HUVECs at CPDL16; RS + NaHS: HUVECs at CPDL16 and exposed to 200 *μ*mol/L NaHS for 24 hours. RS + NaHS + LY:HUVECs at CPDL16 and exposed to 10 *μ*mol/L LY294002 prior to treatment of 200 *μ*mol/L NaHS for 24 hours.

We detected the protein expression in the control group (CPDL2 cells), replicative senescence model group, NaHS intervention group, and PI3K block group to inquire into the involvement of PI3K/Akt/eNOS pathway. As compared with the control group, protein levels of PI3K, Akt, p-Akt, eNOS, and p-eNOS were decreased in the replicative senescence model but restored by NaHS intervention (Figures 7(a)–7(b)). However, PI3K blockade abolished the recurring effect of NaHS, as LY294002 resulted in suppressed expression of the above proteins. In order to prove that NaHS promotes the production of NO in endothelial cells through the PI3K/Akt/eNOS signaling pathway to facilitate vasodilation, we also detected the NO content in the supernatant of each group. As shown in Figure 8, content of replicative senescent cells produced less NO than control cells (CPDL2). NO production was induced in the NaHS intervention group when compared with the replicative senescence model group but restrained in the PI3K blocking group. These results suggest that NaHS might improve the replicative senescence of endothelial cells by activating the PI3K/Akt/eNOS pathway, and treatment with a PI3K inhibitor impeded the regulation of NaHS on the PI3K/Akt/eNOS pathway and impaired the protective effect of NaHS on endothelial cells.

## 4. Discussion

The study established a replicative senescence model of HUVECs by continuous passage, as confirmed by elevated expression of PAI-1 protein and increased positive rate of SA-*β*-Gal staining. However, PAI-1 level and SA-*β*-Gal staining were reduced in NaHS-treated cells, suggesting that exogenous H_2_S successfully delayed the senescence of HUVECs. Mechanistic experiments revealed that NaHS exerted its effects of rescuing the replicative senescence possibly through activating PI3K/Akt/eNOS pathway.

Atherosclerosis among the aging population is closely related to the senescence of vascular endothelial cells [17]. Cellular senescence is classified into replicative senescence and precocious senescence. Induced and precocious cell senescence reflects the senescence phenomenon under stress, which partially simulates the actual process of cell senescence under physiological circumstances. On the other hand, replicative senescence of cells occurs when cultured diploid cells experience repeated rounds of mitosis developed limitations in mitotic capability, characteristic of cell morphological changes, proliferation stagnation, growth arrest, loss of differentiation ability, and significant changes in physiological metabolic activities. Therefore, cellular replicative senescence, given its accordance with the physiological process of human natural aging, attracts growing attention. In our study, cells at CPDL 16 and beyond stably presented positive indicators of cellular senescence. Under microscopic observation, senescent endothelial cells are larger in size with blurred borders and a flat shape, accompanied by over 50% of SA-*β*-Gal staining and significantly upregulated expression of PAI-1. The phenotypic and molecular changes were consistent with the characteristics of cell senescence, indicating that our endothelial cell replicative senescence model was successfully established.

As a gaseous signal molecule in the human body, H_2_S exerts a crucial protective effect on cardiovascular function [18]. It has been reported that H_2_S could postpone the formation of atherosclerotic plaques and reduce the existing plaque. One of the key factors in atherosclerosis is the deduction of H_2_S level [19]. Our previous research also validated that exogenous H_2_S alleviated endothelial cell senescence by overcoming oxidative stress [16] and regulating the Sirt1/eNOS signaling pathway [20]. Another gaseous signal molecule, NO, is mainly produced by its rate-limiting enzyme eNOS in endothelial cells [21] and functions by suppressing apoptosis and inhibiting the adhesion of leukocytes and platelets to the blood vessel wall. It also participates in regulating vascular tension and blood flow distribution, thereby affecting the occurrence and development of cardiovascular and cerebrovascular diseases [22]. NO alone or in synergy with H_2_S can relax vascular smooth muscle; moreover, a single application of H_2_S only arouses a very weak vasodilation effect, which can be amplified in the presence of NO [23].

PI3K is an important lipid kinase that converts phosphatidylinositol-4, 5 diphosphate [PI(4, 5)P_2_] to phosphatidylinositol-3, 4, 5 triphosphate (PIP_3_) by phosphorylating phosphatidylinositol, and PIP_3_ acts as an important secondary messenger in cells, recruiting Akt to the plasma membrane as well as phosphorylating and activating it [24], thereby exerting biological effects. The PI3K/Akt pathway has various functional effects. For instance, some traditional Chinese medicines can alleviate the injury of acute cerebral ischemia and liver ischemia-reperfusion in mice through the PI3K/Akt pathway [25, 26]. PI3K/Akt signaling is also involved in multiple scenarios, including Aurora inhibitors restraining the in vitro proliferation of colon cancer [27] and adiponectin alleviating lipopolysaccharide-induced apoptosis [28]. Akt stands as a central link in PI3K/Akt signaling. Upon phosphorylation and activation, Akt further regulates the activation and expression of various downstream effectors. Typically, phosphorylated Akt not only inhibits apoptosis by increasing the expression of apoptosis regulators [29], but also alleviates the restriction upon the survival of vascular smooth muscle cells by inhibiting PTEN, thereby protecting cardiomyocytes from damage [30]. eNOS is also a key downstream substrate of Akt, which can be directly phosphorylated at Ser177 and then activated by Akt [31]. Activated eNOS can further catalyze the production of No to protect the vascular endothelium [32]. Furthermore, phosphorylation of Akt facilitates vasodilation by enhancing NO release from eNOS through PI3K/Akt/eNOS signaling [33]. Based on PI3K/Akt/eNOS signaling, nicorandil ameliorates Hcy-induced coronary microvascular dysfunction in mice [34], and mouse aortic endothelial cell dysfunction can also be alleviated [35].

Several studies have focused on the relationship between H_2_S and Akt phosphorylation. According to the documentary, H_2_S delays the senescence of HUVECs induced by H_2_O_2_ via PI3K/Akt/eNOS axis [8] and promotes the formation of neovascularization and growth of vascular endothelial cells in the survival rat model, dependently on the activation of the PI3K/Akt cascade within vascular endothelial cells. On the other hand, it is reported that H_2_S prohibits Akt hyperphosphorylation and therefore delays endothelial cell senescence [36]. However, little is known about the physiological function and mechanism of H_2_S in the replicative senescence model of in vitro endothelial cells. Therefore, our study further enriched the mechanism of H_2_S in mitigating endothelial cell senescence, especially replicative senescence.

In our study, we successfully established a replicative senescence model of endothelial cells, as validated by the reduced expression of PI3K, p-Akt, p-eNOS, and NO content. Nonetheless, NaHS treatment resulted in the recovery of protein expression and NO release. The regulatory effects of NaHS were reversed in the presence of LY294002, a specific inhibitor of PI3K. Accordingly, we speculated that NaHS upregulates PI3K/Akt effectors that increase eNOS phosphorylation and consequently induces the intracellular NO content to improve endothelial cell senescence. This study further clarified that the improvement effect of NaHS on endothelial cell senescence is closely associated with PI3K/Akt/eNOS activation. In conclusion, our replicative senescence of endothelial cells, in line with human physiological processes, verifies the feasibility and possible mechanism of exogenous H_2_S in preventing and treating endothelial cell senescence, which provides new ideas and targets for clinical interventions in endothelial cell senescence and atherosclerosis.

## Data Availability

The data used to support the finding of this study are available from the corresponding author on reasonable request.
